# Supplementary material for: Exogenous Administration of Low-Dose Lipopolysaccharide Potentiates Liver Fibrosis in a Choline-Deficient l-Amino-Acid-Defined Diet-Induced Murine Steatohepatitis Model
Source: Int J Mol Sci. 2019 Jun 3;20(11):2724. doi: 10.3390/ijms20112724 (PMC6600174; doi:10.3390/ijms20112724)
Supplement: Supplementary file 1 [file ijms-20-02724-s001.pdf]

International Journal of
Molecular Sciences

MDPI

Supplementary Table1. List of primers used in q-PCR

Gene	Sense (5’-3’)	Antisense (5’-3’)
Lbp	TCGTGGGCAGTACGAGTTTC	AAGAGATTCAGCAGCCACCC
Cd14	GTCAGGAACTCTGGCTTTGC	TGGCTTTTACCCACTGAACC
Srebf1	GATCAAAGAGGAGCCAGTGC	TAGATGGTGGCTGCTGAGTG
Fas	TGGGTTCTAGCCAGCAGAGT	ACCACCAGAGACCGTTATGC
Acc1	GCCTCTTCCTGACAAACGAG	TGACTGCCGAAACATCTCTG
Ppara	GAGGGTTGAGCTCAGTCAGG	GGTCACCTACGAGTGGCATT
Pparg	CTGTGAGACCAACAGCCTGA	AATGCGAGTGGTCTTCCATC
Tnfa	TTCTATGGCCCAGACCCTCA	TGGTTTGCTACGACGTGGG
Il1b	GCCCATCCTCTGTGACTCAT	AGGCCACAGGTATTTTGTCG
Il6	AGTTGCCTTCTTGGGACTGA	TCCACGATTTCCCAGAGAAC
Acta2	CTGACAGAGGCACCACTGAA	CATCTCCAGAGTCCAGCACA
Col1a1	GAGCGGAGAGTACTGGATCG	GCTTCTTTTCCTTGGGGTTC
Tgfb1	TTGCTTCAGCTCCACAGA GA	TGGTTGTAGAGGGCAAGGAC
Gapdh	CCACCCAGAAGACTGTGGAT	CACATTGGGGGTAGGAACAC
